# Automated sample preparation for electrospray ionization mass spectrometry based on CLOCK-controlled autonomous centrifugal microfluidics

**DOI:** 10.1007/s10544-024-00703-4

**Published:** 2024-04-09

**Authors:** Masahiro Futami, Hiroki Naito, Satoshi Ninomiya, Lee Chuin Chen, Tomohiko Iwano, Kentaro Yoshimura, Yoshiaki Ukita

**Affiliations:** 1 Department of Engineering, Integrated Graduate School of Medicine, Engineering, and Agricultural Sciences, Graduate School of University of Yamanashi, 4-3-11 Takeda, Kofu, 400-8510 Japan; 2 Graduate Faculty of Interdisciplinary Research, University of Yamanashi, 4-3-11 Takeda, Kofu, 400-8510 Japan; 3 Department of Anatomy and Cell Biology, Faculty of Medicine, University of Yamanashi, 1110 Shimokato, Chuo, 409-3898 Japan; 4 Division of Molecular Biology, Center for Medical Education and Sciences, Interdisciplinary Graduate School of Medicine, University of Yamanashi, 1110 Shimokato, Chuo, 409-3898 Japan

**Keywords:** Centrifugal microfluidics, Mass spectrometry, Autonomous microfluidic control, Sample preparation

## Abstract

We report a centrifugal microfluidic device that automatically performs sample preparation under steady-state rotation for clinical applications using mass spectrometry. The autonomous microfluidic device was designed for the control of liquid operation on centrifugal hydrokinetics (CLOCK) paradigm. The reported device was highly stable, with less than 7% variation with respect to the time of each unit operation (sample extraction, mixing, and supernatant extraction) in the preparation process. An agitation mechanism with bubbling was used to mix the sample and organic solvent in this device. We confirmed that the device effectively removed the protein aggregates from the sample, and the performance was comparable to those of conventional manual sample preparation procedures that use high-speed centrifugation. In addition, probe electrospray ionization mass spectrometry (PESI-MS) was performed to compare the device-treated and manually treated samples. The obtained PESI-MS spectra were analyzed by partial least squares discriminant analysis, and the preparation capability of the device was found to be equivalent to that of the conventional method.

**Supplementary Information:**

The online version contains supplementary material available at 10.1007/s10544-024-00703-4.

## Introduction

Blood tests can measure a wide variety of biomarkers for disease and play a major role in clinical testing (Lenders et al. 2014; Sacks et al. 2011). However, generally, in blood tests, the target substance is determined according to the tested item, and substances of no interest are not measured. Therefore, these tests are not able to detect diseases that are not covered by the testing method.

On the other hand, metabolomics research has led to the discovery of disease-associated low-molecular-weight metabolites (Zhang et al. 2012; Mishur and Rea 2012; Wu et al. 2022), and mass spectrometry methods such as electrospray ionization (ESI) have attracted attention as testing technologies (Wu et al. 2022; Nishiumi, et al. 2012; Chung et al. 2020; Johno et al. 2018; Jongejan et al. 2020; Caron et al. 2021). Because mass spectrometry can comprehensively measure the components of a sample, it is expected to be utilized as a novel testing technology for collectively assessing the risk of multiple diseases. However, high concentrations of proteins in the blood cause severe ion suppression due to the electrospray ionization of low-molecular-weight metabolites; thus, the excess proteins need to be removed by treating the tested samples with organic solvents or ultrafiltration (Michopoulos et al. 2009; Zelena et al. 2009). These preparatory steps include purification processes involving reagent mixing, agitation, and centrifugation and require many instruments and complicated operational workflows; consequently, their high cost and long processing time are challenging for high-throughput clinical applications such as point-of-care testing. Therefore, low-cost, fast, and simple technologies need to be developed for processing samples to enable mass spectrometry-based testing.

Microfluidic devices have various advantages, such as automation and faster chemical processing, owing to trace amounts and faster reactions (Lai et al. 2004; Nge et al. 2013). Consequently, the use of microfluidic devices for sample sampling and processing before analysis by mass spectrometry (MS) has been investigated (Luk et al. 2009, 2012; Jebrail et al. 2011; Shih et al. 2012; Gustafsson et al. 2004; Zhao et al. 2019). However, these studies suggested the need for complex and sophisticated fluidic controls. For example, digital microfluidics-based devices (Luk et al. 2009, 2012; Jebrail et al. 2011; Shih et al. 2012) use electrowetting for fluid control, and electrowetting has a complex device structure and expensive manufacturing process. Pre-MS sample preparation with centrifugal microfluidic devices has also been reported (Gustafsson et al. 2004; Zhao et al. 2019); however, columns and filters had to be implemented, complicating the device structure and manufacturing process.

In this study, we report the development of an automated preparation device. We devised a novel sample preparation device based on a unique and simple centrifugal fluid control technique; this technique is called the control of liquid operation on centrifugal hydrokinetics (CLOCK) (Okamotoa and Ukita 2017). This device features a water clock with microchannels and a siphon; the injection order and timing of reagents and organic solvents are controlled by steady rotation. In addition, a novel reagent-agitation mechanism combined with a unique pneumatic control mechanism was designed. The remainder of this article is organized as follows. First, we describe the concept of the automated preparation device and demonstrate our proposed method. Next, we examined the reagent-agitation mechanism implemented in the device and evaluated its mixing performance and reproducibility. The removal rates of protein aggregates (debris) generated during device operation were compared with those of standard centrifugation and supernatant decanting techniques. Finally, the mass spectra of the samples obtained using our proposed device are compared with those of the samples obtained by manual preparation for evaluation of the performance of our proposed device.

## Materials and methods

### Protocol and design of the device

When measuring low-molecular-weight metabolites, high-concentration proteins need to be removed. In this process, the tested sample is first added to an organic solvent, mixed, diluted, and then allowed to stand at room temperature to enable the high-molecular-weight components to aggregate and precipitate as debris. The debris is then separated by centrifugation, and the extracted supernatant is analyzed by electrospray ionization mass spectrometry (ESI–MS). We designed a device mechanism that automatically performs these processes.

**Fig. 1:**
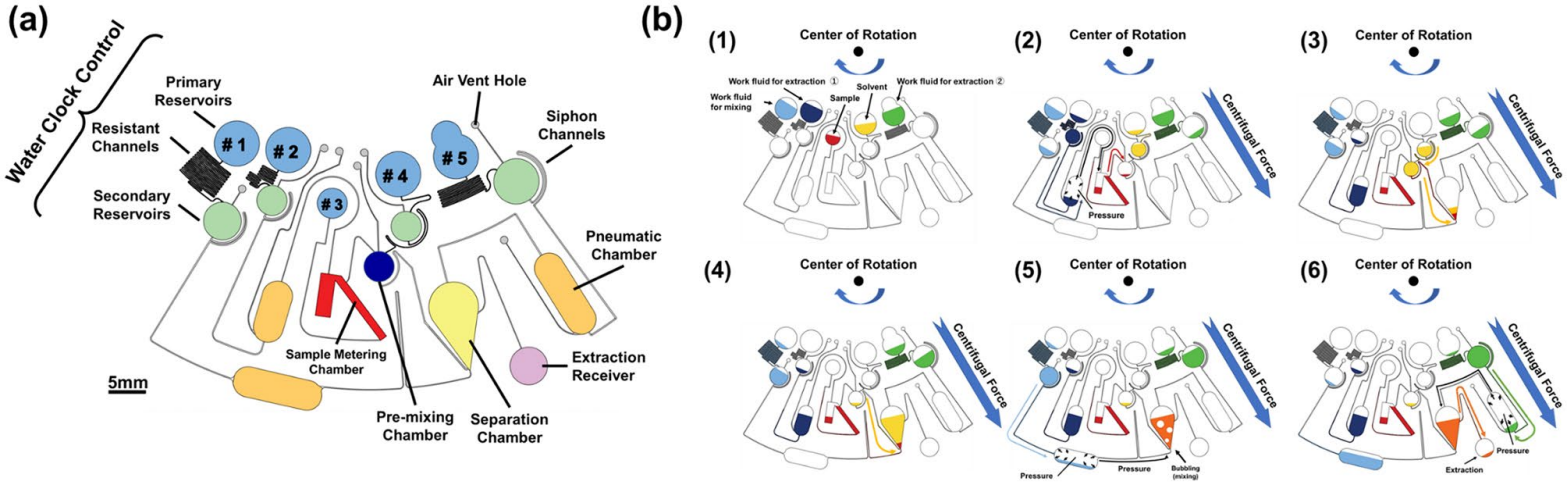
Schematic of the proposed device. **a** Design of the proposed device. **b** Schematic of the device operation: (1) initial state, (2) sample extraction, (3) injection of the extraction solvent, (4) injection into the separation chamber, (5) mixing, and (6) supernatant extraction

**Fig. 2:**
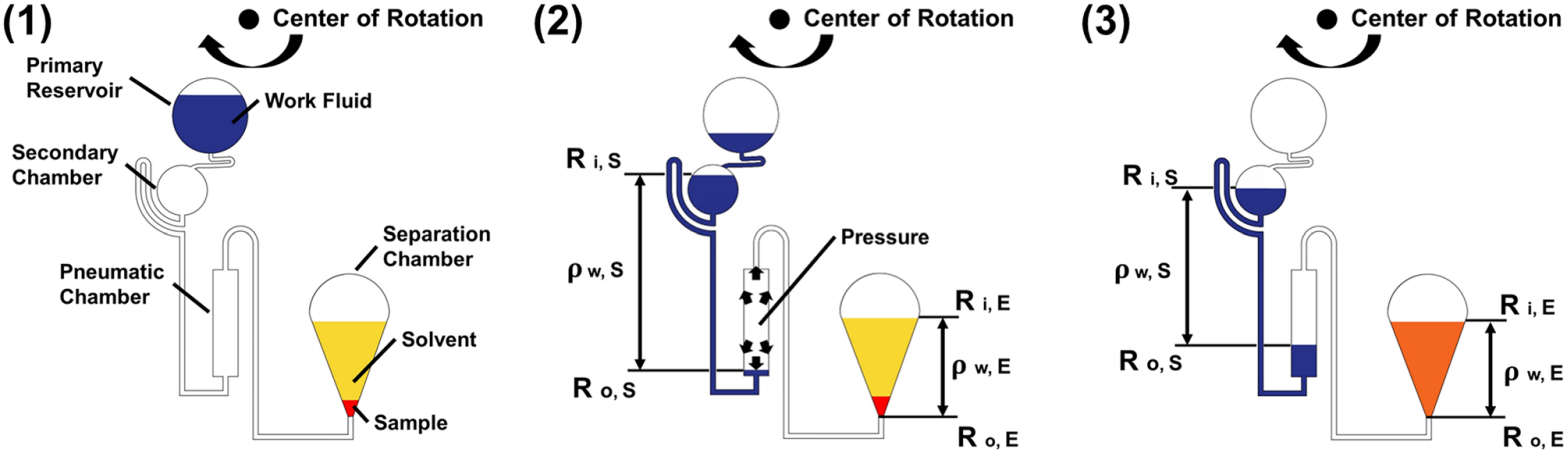
Schematic of the agitation mechanism. (1) Initial state and (2) start of agitation. As the work fluid is injected into the pneumatic chamber, the pressure in the pneumatic chamber increases, and air flows into the separation chamber. (3) Termination condition of the mechanism

**Fig. 3:**
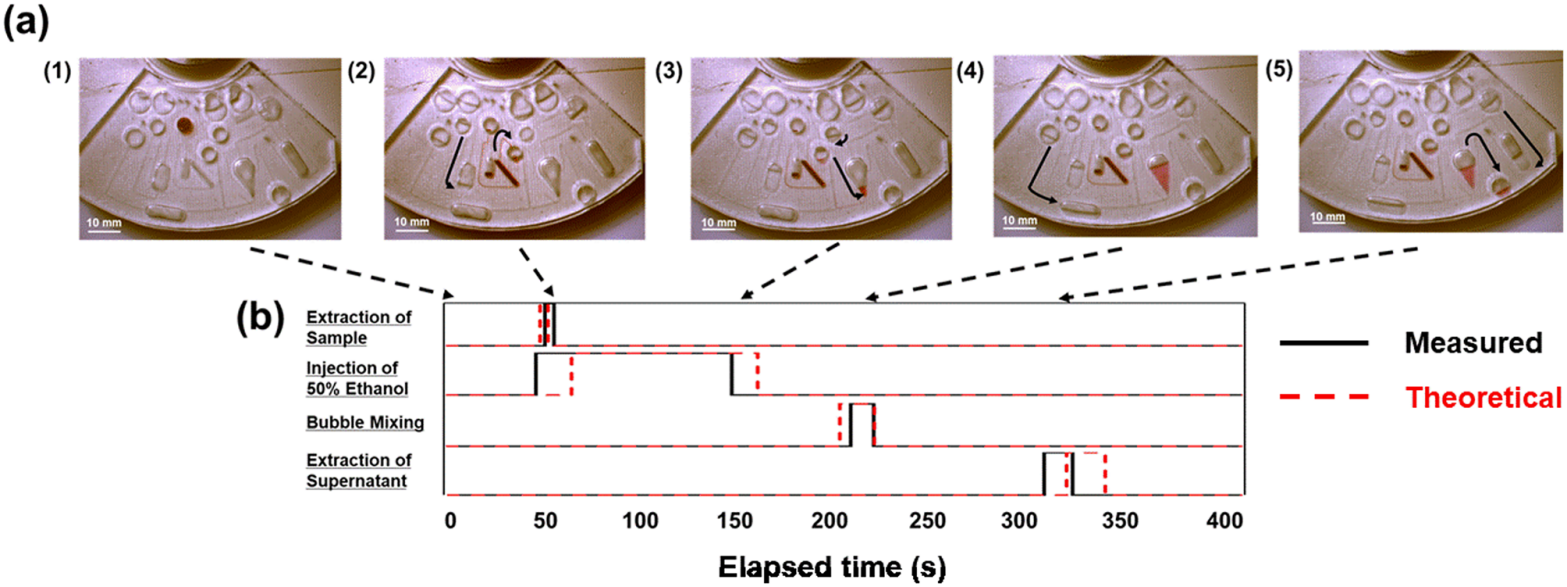
Time chart and photographs of the device operation. **a** Photographs of the device operation: (1) initial state, (2) sample extraction, (3) injection into the separation chamber, (4) mixing, and (5) supernatant extraction. **b** Time chart of the device operation. The solid lines indicate the measured values, and the dashed lines indicate the theoretical values of the device operation

**Fig. 4:**
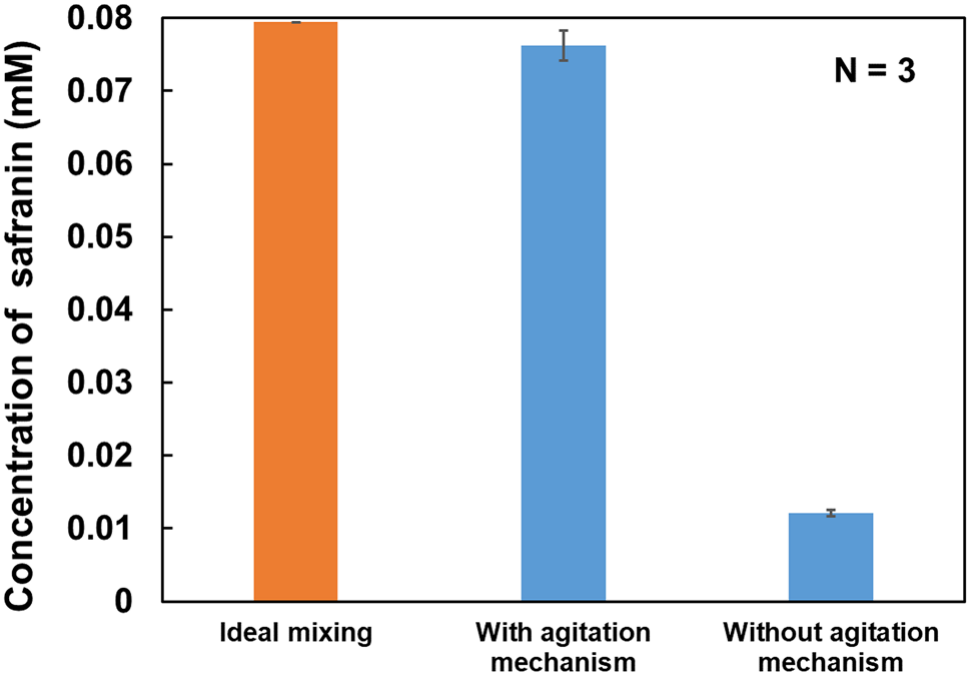
Theoretical sample concentration and average sample solution concentration. The absorbance of the sample solution at 520 nm was measured. The sample solution concentrations were calculated using the absorbance at 520 nm of the mock samples diluted 20, 40, 80, 160, and 320 times with the extraction solvent as standards. Error bars are the standard deviations

**Fig. 5:**
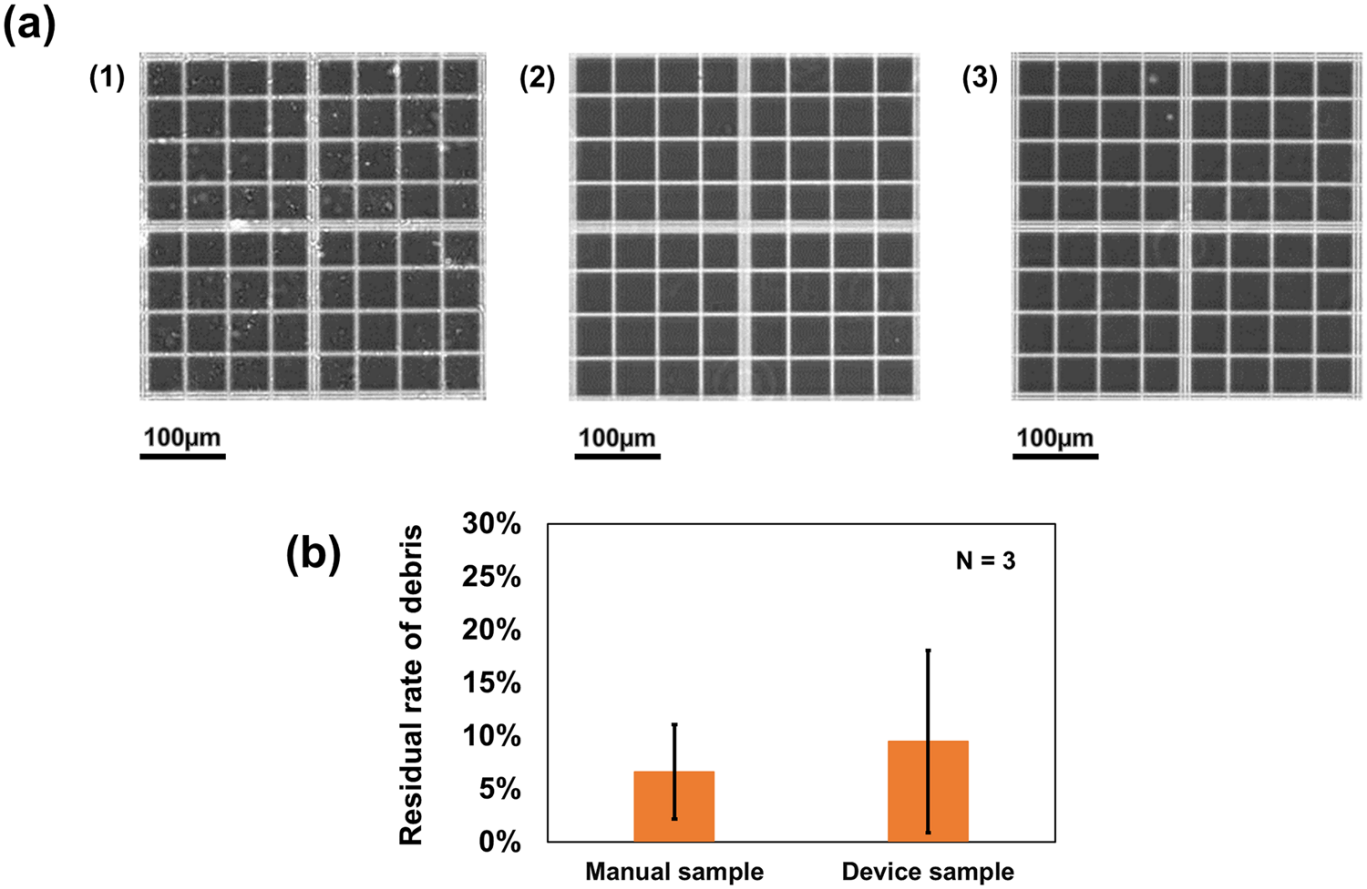
Comparison of the manual and device debris removal capabilities. **a** Comparison of the debris removal performance for different treatment conditions: (1) without centrifugation, (2) manual operation, (3) device-based operation, and (**b**) without the average debris residual rate using centrifugation as the reference. Error bars indicate the standard deviations

**Fig. 6:**
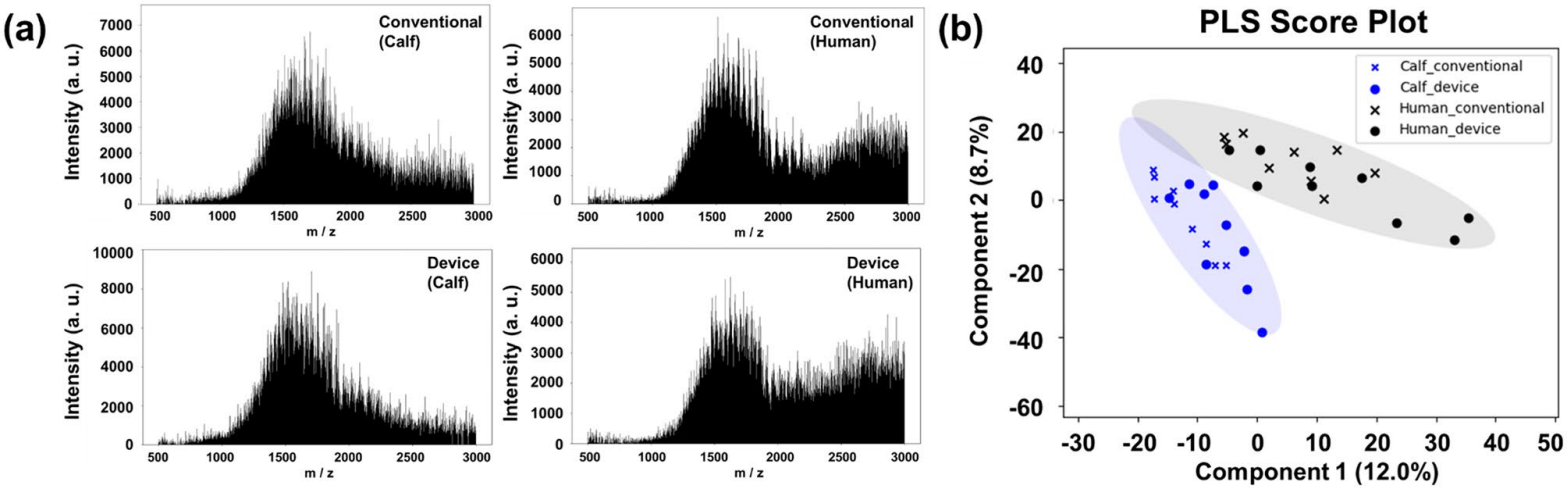
Comparison of the manual and device-based preparation capacities. **a** Mass spectra measured using PESI-MS. **b** Results from the PLS-DA and k-means analyses

Figure 1(a) shows the structure of the proposed device. The device consists of the following: primary reservoirs for sample injection, resistant channels, and secondary reservoirs to adjust the timing of the reagent injection; a metering chamber for measuring the sample volume to be pretreated; pneumatic chambers for sample extraction and mixing; a separation chamber for removing debris from the sample after dilution; and a supernatant chamber for holding the pretreated sample. Figure 1(b) schematically shows the device operation. As shown in Fig. 1(b-1), the reagents are injected into each primary reservoir before the start of rotation. After the rotation is started, the reagent injected into primary chamber #3 is immediately injected into the sample-metering chamber. At this time, the excess sample overflows, and the sample is metered. The secondary reservoir downstream of #2 is then filled, and the work fluid passes over the siphon and is injected into the pneumatic chamber, which is located further downstream. Pressure is applied to the sample in the sample-metering chamber. The generated pressure causes the metered sample (approximately 0.5 μL) to be injected into the premixing chamber from the sample-metering chamber (Fig. 1(b-2)). This timing is adjusted by the CLOCK mechanism, which is composed of a resistant channel and a secondary chamber that are able to wait for the completion of sample metering. Next, the secondary reservoir downstream of #4 is filled, and the organic solvent passes over the siphon and is injected into the premixing chamber (Fig. 1(b-3)). After the premixing chamber is filled with the organic solvent, the sample/solvent mixture is injected into the separation chamber (Fig. 1(b-4)). Next, the secondary reservoir downstream of #1 is filled, and the work fluid passes over the siphon and is injected into the pneumatic chamber further downstream. The solution in the separation chamber is mixed by bubbling (Fig. 1(b-5)). After mixing, the solution remains in the separation chamber, and the agglomerated debris is centrifuged. Finally, after filling the secondary chamber downstream of #5, the work fluid is injected over the siphon into the pneumatic chamber further downstream, applying pressure to the solution in the separation chamber. In this method the purified sample solution is injected into the extraction receiver (Fig. 1(b-6)).

The reagent and supernatant extraction mechanisms of the proposed device are based on previously published methods (Naito et al. 2018). The agitation mechanism by bubbling is completed using the CLOCK circuit and the hydraulic head pressure of the liquid. Figure 2 shows the steps involved in the agitation process. Figure 2 (1) shows the initial state of the agitation process. Figure 2(2) shows the work fluid over the siphon. Because the hydraulic head pressure of the work fluid is greater than that of the liquid in the separation chamber, the air in the pneumatic chamber is injected into the separation chamber, and the sample and solvent are agitated by bubbling. The conditional expression for bubbling is as follows:

1 $${\uprho }_{{\text{w}},{\text{S}}}\left({{{\text{R}}}^{2}}_{{\text{o}},\mathrm{ S}} -{{{\text{R}}}^{2}}_{{\text{i}},\mathrm{ S}}\right) > {\uprho }_{{\text{w}},{\text{E}}}\left({{{\text{R}}}^{2}}_{{\text{o}},\mathrm{ E}} -{{{\text{R}}}^{2}}_{{\text{i}},\mathrm{ E}}\right)$$

where ${\uprho }_{{\text{w}},{\text{S}}}$ is the mass density of the work fluid, and ${\uprho }_{{\text{w}},{\text{E}}}$ is the mass density of the liquid in the separation chamber. ${{\text{R}}}_{{\text{o}},\mathrm{ S}}$ and ${{\text{R}}}_{{\text{i}},\mathrm{ S}}$ denote the outer and inner radial positions of the work fluid in the agitation mechanism, respectively. ${{\text{R}}}_{{\text{o}},\mathrm{ E}}$ and ${{\text{R}}}_{{\text{i}},\mathrm{ S}}$ denote the outer and inner radial positions, respectively, of the fluid in the separation chamber. As the work fluid in the secondary chamber flows into the pneumatic chamber, the hydraulic head pressure of the work fluid decreases and reaches equilibrium with the hydraulic head pressure of the liquid in the separation chamber, and the agitation is terminated (Fig. 2(3)).

### Device fabrication procedure

All tested devices were fabricated using the soft lithography method (Duffy et al. 1998). Device molds were prepared by spin-coating a photoresist (SU-8 3050, Nippon Kayaku Co., Ltd., Japan) onto a 4-inch silicon wafer at a thickness of approximately 105 µm and soft-baked at 95 °C for 45 min. The channel pattern drawn on a photomask (width, 100 µm) was transferred to the photoresist by ultraviolet (UV) exposure. The substrate was post-baked at 95 °C for 5 min and developed using a developer (SU-8 Developer; Nippon Kayaku Co.). Chamber molds, which were made as described below, were placed on the developed device molds and baked at 85 °C using a hot plate for bonding. Liquid polydimethylsiloxane (PDMS) mixed with a PDMS monomer and cross-linker at 10:1 (wt: wt) was applied at a thickness of 2 mm and cured by baking in an oven at 75 °C for 90 min. The PDMS sheet on which the channel structure was transferred (main PDMS sheet) was peeled off from the mold, the external shape was prepared with a cutter, and the chambers and vents were punched. The main PDMS sheet and the 1-mm-thick PDMS sheet were ultrasonically cleaned with 99.5% ethanol (99.5%, Kanto Chemical Co., Inc., Japan) and Milli-Q water for 5 min, followed by annealing in an oven at 200 °C for 30 min. After cooling, a plasma cleaner (YHS-R, SAKIGAKE-Semiconductor Co., Ltd., Japan) was used to bond the top surface of the main PDMS sheet to a 1-mm-thick PDMS sheet by surface-activated bonding. Vent inlets were formed by punching holes in the bonded PDMS sheet. To seal the flow channel, the main PDMS sheet was bonded to a 1-mm thick PDMS sheet by surface-activated bonding using a plasma cleaner. The master pattern of the chamber molds was made of carving wax (File-A-Wax, Freeman Mfg. & Corp., U.S.A.), machined based on 3D CAD data and bonded to a silicon wafer at 108 °C. Then, PDMS was applied (thickness, 2 mm) and cured by baking at 75 °C for 90 min in an oven. The PDMS sheet, in which the carving wax shape was transferred, was peeled off from the wafer. Heated hot-melt glue (02280242, MonotaRO Co., Ltd., Japan) was poured into the chamber-shaped cavity of the PDMS sheet, pressed with an OHP film (VF-1410N, KOKUYO Co., Ltd., Japan), and allowed to naturally cool to room temperature. The hardened glue was removed from the PDMS sheet, and the outer shape was prepared using a cutter to create a chamber mold.

### Reagent preparation

The extraction solvent was prepared by mixing anhydrous ethanol (99.5%, Wako Pure Chemical Industries, Ltd.) and ultrapure water (Wako Pure Chemical Industries, Ltd., Japan) at a ratio of 1:1 (v/v). Milli-Q water was used as the work fluid. For the mock samples that were used for validating the device behavior and the agitation mechanism of reagents, 0.2 wt% safranin (Wako Pure Chemical Industries, Ltd., Japan) was added to Milli-Q water, vortexed for 5 min, sonicated for 5 min, and then filtered through a syringe filter (Nylon Syringe Filter, Membrane Solutions, USA). Human serum (human serum, Cosmo Bio Co., Ltd., Japan) and neonatal bovine serum (Newborn calf serum, Thermo Fisher Scientific Co.) were used as the evaluation samples.

### Sample preparation procedure

The experiments were performed according to the following procedure. Fifteen microliters of the sample was loaded into the #3 primary reservoir. Fifty microliters of the work fluid was loaded into the #1 and #2 primary reservoirs, while 53 µL was loaded into the #5 primary reservoir. Thirty microliters of the extraction solvent was injected into the #4 primary reservoir. The device was then mounted on a rotating device (Ukita and Takamura 2015), rotated at 1500 rpm, and maintained for 500 s at an acceleration of 1500 rpm. After the rotation was completed, 10 µL of sample solution was collected in a tube. The behavior of the liquid during processing was recorded using a stroboscope (Grumann et al. 2005). The conventional method involved the following steps: Ten microliters of the sample was added to 710 µL of the extraction solvent and mixed for 30 s. After 10 min of remaining at room temperature, the sample was centrifuged at 17,700 × g for 2 min. The supernatant (72 µL) was collected in a tube and used as the sample solution.

### Mass spectrometry and data handling for multivariate analysis

Because the sample solution treated by the preparation device did not have sufficient volume for multiple measurements, it was diluted tenfold with the extraction solvent to enable multiple measurements. To ensure uniform conditions, the sample solutions treated by the conventional method were diluted tenfold with the extraction solvent. The assay solution was prepared by adding acetic acid (99.8%, Wako Pure Chemical Industries, Ltd., Japan), which was equivalent to 1% of the sample volume, to the reagent solution diluted tenfold with the extraction solvent. The solution (20 µL) was placed on a sample holder for analysis by PESI-MS (Hiraoka et al. 2007) using a commercial mass spectrometer (Exactive Plus Orbitrap Mass Spectrometer, Thermo Fisher Scientific, USA). The original ion interface of the mass spectrometer was modified to accommodate the custom-made PESI ion source. The PESI ion source consisted of a solid needle that served as the sampling probe and an ESI emitter (J-Type Acupuncture Needle No. 02 × 40 mm, SEIRIN Co., Japan). The probe needle was attached to a motorized linear actuator (SCN5-010–050-s03, Dyadic Systems Co.) that moved up and down along the axis perpendicular to the sample surface. The highest position of the needle tip was the ionization position, and the lowest position was the sampling position. The sample stage was positioned below the ion sampling orifice. When the probe needle was at the sampling position, the tip was dipped into the sample solution at an adjustable depth. The adhered solution was then moved to the ionization position for electrospray ionization (Yoshimura et al. 2011). The probe needle was electrically connected to the ground at the sampling position, and when it moved to the ionization position, a high voltage (1.6–1.9 kV) was applied from a DC power supply (HJPM-5R1.2, Matusada Precision Inc., Japan). The switching between the HV and Gnd was accomplished using a push–pull transistor switch (HTS61-03-GSM 2 × 6 kV/30A, Behlke, Germany), and the timing was synchronized with the needle motion using a digital delay and pulse generator (DG535, Stanford Research Systems, USA). The frequency of the needle movement was 1 Hz. After 50 ms of sampling, the needle was moved to the ionization position, and after 150 ms, voltage was applied for 500 ms. The mass spectral data were arranged to have 225,000 mass signals in the m/z range of 500–3000 (the intensity of the non-measured mass signal was 0). The mass spectra for each measured solution were subjected to partial least squares discriminant analysis (PLS-DA) (Barker and Rayens 2003).

## Results and discussion

### Demonstration of the automated preparation

The operation of the device on a mock sample is shown in Fig. 3(a) and Supplementary Movie 1. The liquids were held in the primary reservoirs before applying the centrifugal force (Fig. 3(a-1)). The centrifugal force was applied to inject and meter a sample from #3 into the sample-metering chamber. Fifty-five seconds after the rotation started, the work fluid was injected from the secondary reservoir downstream of #2 into the pneumatic chamber further downstream, and the mock sample was injected into the premixing chamber (Fig. 3(a-2)). At 160 s after the start of rotation, the premixing chamber was filled with the extraction solvent and injected into the separation chamber (Fig. 3(a-3)). At 220 s after the start of the rotation, the work fluid was injected from the secondary reservoir downstream of #1 into the pneumatic chamber further downstream, and bubbling was performed (Fig. 3(a-4)). At 304 s after the start of rotation, the work fluid was injected from the secondary reservoir downstream of #5 into the pneumatic chamber further downstream, and the supernatant of the sample solution was injected into the extraction receiver (Fig. 3(a-5)). Figure 3(b) shows a comparison of these injection timings with the theoretical values. The time at which the extraction solvent from the premixing chamber was injected into the separation chamber occurred slightly earlier than the theoretical value, and the extraction solvent was already present in the premixing chamber before the injection of the mock sample; therefore, the operation procedures slightly differed from those of the original design. The coefficient of variation (CV) of the time for each step was less than 7% after 6 replicate experiments, indicating sufficient stability. The injection timing of the extraction solvent was approximately 20% earlier than the theoretical value, whereas this phenomenon was not observed for the work fluid. We confirmed that the wettabilities of the extraction solvents and work fluids with respect to PDMS were 71° and 110°, respectively. Therefore, the extraction solvent was transported into the resistance channel by capillary action before rotation due to its higher wettability. In our experiments, the extraction solvent was at the junction between the resistance channel and the secondary reservoir and was immediately injected into the secondary reservoir after the start of rotation. This phenomenon must be considered when rigorous design is needed. As described above, automatic pretreatment during steady rotation was demonstrated by combining the CLOCK paradigm and the pneumatic mechanism. Compared to other proposed pneumatic mechanisms (Hess et al. 2019), our proposed pneumatic mechanism requires a work fluid, and the number of chambers are increased; however, a working device can be simply developed by sealing the molded channel structure. This will lead to improved device productivity and reduce product production costs. Since the device is operated with steady rotation, its control system can be simplified, and it will likely contribute to improving the operability of the device, reducing the cost of the entire system, and producing a more compact system. This would be a valued feature even in medical settings where time and space resources are limited.

### Evaluation of sample mixing

The mixing performance of the developed device was evaluated based on the concentration of safranin added to the mock sample. This was verified by comparing the results obtained using the agitation mechanism with the results obtained without using the agitation mechanism. Therefore, 30 µL (without the agitation mechanism) or 50 µL (with the agitation mechanism) of the work fluid was injected into primary reservoir #1 to achieve the desired conditions. The conditions for the injection and rotation of reagents into the remaining primary reservoirs are described in Section 2.4. The extracted sample was collected, and the absorbance at 520 nm was measured using a spectrophotometer (NanoDrop One, Thermo Fisher Scientific) to evaluate the effect of the agitation mechanism with respect to the concentration of safranin. Figure 4 shows the concentration of safranin under three different conditions: 1) ideal mixing, 2) without the agitation mechanism, and 3) with the agitation mechanism. The concentration of the sample solution without the agitation mechanism was 0.012 mM, whereas the average concentration of the sample solution with the agitation mechanism was 0.076 mM, showing a significant difference between the two results. This difference was caused by not using the agitation mechanism; the reagent was not agitated, and the densities were different between the extraction solvent and the mock sample. In centrifugal microfluidics, a small density difference (0.05 g/cm^3^) could create a stable boundary layer in the liquid, and spontaneous mixing would not occur (Ukita et al. 2017). In our system, the buffer density of the mock sample (Milli-Q water) was 0.999 g/cm^3^, whereas the extraction solvent density was 0.93375 g/cm^3^. Therefore, spontaneous mixing did not occur. On the other hand, the theoretical concentration and the concentration for the scenario in which the agitation mechanism was used were close to each other. The CV values of the sample solution were less than 3% when the agitation mechanism was used and less than 4% without the agitation mechanism. Based on the above results, we confirmed that our mechanism was effective for reagent agitation. Several studies have been conducted to achieve bubbling via gas pressurization. On the other hand, the methods for reagent agitation by bubbles developed thus far use additional elemental technologies, such as external devices (Veres et al. 2015) and catalysts (Burger et al. 2016), to control the timing of the start of agitation and to generate bubbles. On the other hand, our proposed mechanism attains timing control and bubbling under a steady-state rotation using CLOCK and the water head pressure. This mechanism achieves reagent agitation based on the structure of the microchannel, such as the microchannel length and chamber position, and can be driven and controlled using only the centrifugal force generated by a simple motor. Therefore, effective mixing can be achieved using this low-cost system.

### Evaluation of sample preparation

The sample preparation was evaluated by comparing the results from the debris removal capability and discrimination by mass spectrometry with those obtained using conventional manual methods. First, the debris removal capacity of the device was verified. Figure 5(a) shows the results of the phase-contrast observations for the different sample solutions. The solution that was prepared without centrifugation was the supernatant of the sample solution that was allowed to stand for 10 min before centrifugation using the conventional method (Fig. 5(a-1)). The sample labeled “manual operation” was the sample solution prepared using the conventional method (Fig. 5(a-2)). The sample labeled “device-based operation” was the sample solution prepared using our proposed device (Fig. 5(a-3)). From Fig. 5(a), the “manually prepared” and “device” samples clearly feature less debris than the “without centrifugation” samples. Figure 5(b) shows the percentage of residual debris calculated by visually counting the debris in each sample. Based on the operation conditions and device geometry, the equivalent centrifugal gravity applied and the centrifugal time were 157 × g and approximately 80 s, respectively, while those of the conventional methods were 17,700 × g and 10 min. Even though there was a significant difference between the centrifuge conditions, no significant difference was observed between the average removal rates, with 94% (CV, 47%) for the manual method and 91% (CV, 67%) for the device method. Next, we examined the discrimination using mass spectrometry. This was accomplished by performing mass spectrometry analysis of the sample solutions pretreated with conventional or device-based methods. Figure 6(a) shows the mass spectrum of a sample solution containing human serum and bovine neonatal serum. No significant differences were identified. To obtain a scatter plot of the sample clustering, PLS-DA was performed using only the PESI-MS mass spectra of the measured solutions. K-means clustering was performed, with the number of clusters set to 2. Figure 6(b) shows the results from this analysis. The ellipses covering the cluster groups denote 95% confidence intervals. Human serum and bovine neonatal serum samples formed different clusters; based on the trends in the PESI-MS mass spectra and the PLS-DA results, the sample solutions prepared using the conventional and device-based methods were found to be comparable to each other based on our analysis. These results indicate that the developed device can achieve the same results as conventional manual liquid-handling preparation operations.

## Summary

We designed and manufactured a centrifugal microfluidic device for automated mass spectrometry. This device was designed and developed based on the CLOCK concept. The device controls the injection, mixing, and extraction of reagents using a siphon and secondary reservoir that controlled the timing of the liquid-associated operations. The most important feature of the device was the implementation of agitation using pneumatic control during the steady-state rotation.

Evaluation of the performance of the fluid control mechanism confirmed that reagent injection, reagent mixing, and supernatant extraction were automated. Furthermore, the newly developed reagent agitation mechanism exhibited excellent mixing performance and reproducibility, based on the absorbance and standard curve analyses. This agitation mechanism was implemented by controlling the agitation start time and the bubble generation with steady rotation and by devising chip structures such as microchannels and chambers. The sample preparation performance was evaluated by comparing the debris removal capacity and mass spectrometry discrimination with those of conventional manual methods. Using commercially available human serum for debris removal, we confirmed that the debris removal capacity of the device was equivalent to that of the conventional method. Furthermore, we conducted mass spectrometry analysis with the commercial human serum and commercial bovine neonatal serum samples treated using the conventional method or the device-based method. We confirmed that the device-based method had the same preparation capacity as the conventional method. Therefore, the development of a simple preparation device was achieved and comparted to conventional preparation for mass spectrometry; conventional preparation required complicated operations and equipment.

### Supplementary Information

Below is the link to the electronic supplementary material.

Supplementary file1 (MP4 8963 kb)

## Data Availability

All data will be provided upon reasonable request.
